# Spatial Distribution of Podoconiosis in Relation to Environmental Factors in Ethiopia: A Historical Review

**DOI:** 10.1371/journal.pone.0068330

**Published:** 2013-07-09

**Authors:** Kebede Deribe, Simon J. Brooker, Rachel L. Pullan, Asrat Hailu, Fikre Enquselassie, Richard Reithinger, Melanie Newport, Gail Davey

**Affiliations:** 1 Brighton and Sussex Medical School, Falmer, Brighton, United Kingdom; 2 School of Public Health, Addis Ababa University, Addis Ababa, Ethiopia; 3 Kenya Medical Research Institute-Wellcome Trust Research Programme, Nairobi, Kenya; 4 Faculty of Infectious and Tropical Diseases, London School of Hygiene & Tropical Medicine, London, United Kingdom; 5 School of Medicine, Addis Ababa University, Addis Ababa, Ethiopia; 6 International Development Group, Research Triangle Institute, Washington, D. C., United States of America; Kenya Medical Research Institute - Wellcome Trust Research Programme, Kenya

## Abstract

**Background:**

An up-to-date and reliable map of podoconiosis is needed to design geographically targeted and cost-effective intervention in Ethiopia. Identifying the ecological correlates of the distribution of podoconiosis is the first step for distribution and risk maps. The objective of this study was to investigate the spatial distribution and ecological correlates of podoconiosis using historical and contemporary survey data.

**Methods:**

Data on the observed prevalence of podoconiosis were abstracted from published and unpublished literature into a standardized database, according to strict inclusion and exclusion criteria. In total, 10 studies conducted between 1969 and 2012 were included, and data were available for 401,674 individuals older than 15 years of age from 229 locations. A range of high resolution environmental factors were investigated to determine their association with podoconiosis prevalence, using logistic regression.

**Results:**

The prevalence of podoconiosis in Ethiopia was estimated at 3.4% (95% CI 3.3%–3.4%) with marked regional variation. We identified significant associations between mean annual Land Surface Temperature (LST), mean annual precipitation, topography of the land and fine soil texture and high prevalence of podoconiosis. The derived maps indicate both widespread occurrence of podoconiosis and a marked variability in prevalence of podoconiosis, with prevalence typically highest at altitudes >1500 m above sea level (masl), with >1500 mm annual rainfall and mean annual LST of 19–21°C. No (or very little) podoconiosis occurred at altitudes <1225 masl, with annual rainfall <900 mm, and mean annual LST of >24°C.

**Conclusion:**

Podoconiosis remains a public health problem in Ethiopia over considerable areas of the country, but exhibits marked geographical variation associated in part with key environmental factors. This is work in progress and the results presented here will be refined in future work.

## Introduction

Podoconiosis (endemic non-filarial elephantiasis) is a non-infectious geochemical disease arising in barefoot subsistence farmers who are in long-term contact with irritant red clay soil of volcanic origins. The disease causes progressive bilateral swelling of the lower legs. Mineral particles absorbed through the skin are taken up into macrophages into the lymphatic system and result in an inflammatory process leading to fibrosis and obstruction of the vessels. This leads initially to swelling of the foot and the lower leg, which may with time progress to elephantiasis: gross lymphoedema with mossy and nodular changes of the skin [1], [2], [3]. Due to its symptomatology, podoconiosis has a significant economic impact on affected populations: productivity loss for a patient can amount to 45% of total working days per year, with significant associated loss of income [4] In addition, the disease is known to cause considerable social stigmatization [5]. (Figure S1).

Podoconiosis is widely distributed in three continents; Africa, Central America and Asia particularly India [6], [7]. In Africa, at least 10 countries with the disease have been identified including; Ethiopia, Kenya, Tanzania, Uganda, Rwanda, Burundi, Sudan, Equatorial Guinea, Cameroon, Sao Tome and Principe, and the Cape Verde islands [2], [8], [9], [10], [11], [12]. Historically, the disease is thought to have been prevalent in Northern Africa (Algeria, Tunisia, Morocco and the Canary Islands) and Europe (France, Ireland and Scotland); however, due to widespread use of shoes, the disease is no longer found in these areas [6]. Previous studies have documented the association of the disease with irritant red clay soils, which are generated in areas at >1500 metres above sea level (masl), with >1000 mm annual rainfall and maximum temperatures of >20°C [1]. Across tropical Africa, the majority of affected individuals are famers, who have long term contact with the red clay soil [13]. (Figure S2).

The prevalence of the disease varies from country to country. Earlier nationwide surveys documented an average prevalence of 1% (range: 0% to 2.07%) in Burundi [14] and 0.6% (range: 0.1% to 1.7%) in Rwanda [14]. In Ethiopia, prevalence estimates from 56 market counts ranged from 0.4% to 3.7% [15]; these rates are probably underestimates attributable to reduced mobility of patients and stigma. More recent studies in Ethiopia estimated a prevalence of 5.5% in Southern Ethiopia [16], 5.2% in western Ethiopia [17], 7.4% in central Ethiopia [18] and 3.3% in northern Ethiopia [19]. The presence of significant geographical variation in the prevalence of podoconiosis in Ethiopia and other tropical African countries suggests the existence of spatial patterns to disease distribution.

Without doubt, podoconiosis is one of the neglected tropical diseases (NTDs) with the greatest potential for elimination as a public health problem [20], [21]: it is preventable if shoes are consistently worn, and early stages can be successfully treated using a simple lymphoedema regimen [22]. While one million people are estimated to be affected with podoconiosis in Ethiopia [7], and a further 11 million to be at risk [23], control efforts are hampered by a lack of information on geographical distribution. Aside from isolated studies [16], [17], [18], [19], [21], [24], [25], [26], the only previous mapping of this disease was based on market and school surveys in the 1970s [15], [26], [27]. Except for a few observational studies conducted in the early 1970s, there have been no systematic studies to identify the environmental correlates of podoconiosis [1], [2].

However, various relevant environmental observations have been made. In 1984, Price noted that non-filarial elephantiasis occurred in areas where soil originated from volcanic rocks (including basalt) at altitudes greater than 1000 masl [2], and that the prevalence of podoconiosis decreased significantly at the limit of the red soil, declining to almost zero 25 km from the edge of these soils [28]. Meanwhile, Frommel *et al*. described high concentrations of the trace elements zirconium and beryllium in high prevalence areas of southwest Ethiopia [29].

In recent years, robust and practical approaches to mapping have been developed for a range of diseases [30], [31], [32], [33], [34]. Mapping the distribution of NTDs such as onchocerciasis, lymphatic filariasis, schistosomiasis and soil transmitted helminths has led to successful targeting of control measures to areas of greatest need [30], [31], [32], [33], [34], [35]. With the recent availability of new technologies such as remote sensing and geographic information systems along with advances in spatial and temporal statistics, the theories of landscape ecology can be used analytically [36], [37]. In relation to podoconiosis, the presence of certain environmental factors conducive to the production of irritant soil may be mapped and used to predict the extent of podoconiosis. If interventions for the treatment and prevention of podoconiosis are to be scaled up, information on the distribution and extent of the disease must be available to decision makers. The objective of this study was therefore to investigate the spatial distribution and ecological correlates of podoconiosis using historical and contemporary survey data.

## Methods

### Data Source and Abstraction

The bibliographic databases of MEDLINE (http://medline. cos.com/), EMBASE (http://www.embase.com/) and PubMed (http://www.ncbi.nlm.nih.gov/pubmed/) were searched for relevant English language studies. For identification of publications on podoconiosis, the following Medical Subject Headings (MeSHs) were used to identify relevant studies published between 1960 and 2012: podoconiosis, non-filarial elephantiasis and Ethiopia. Abstracts of studies presenting prevalence were reviewed and full texts retrieved if they contained relevant information. References from articles and key reviews were screened for additional studies. Finally, leading researchers in the area and authors of key papers were contacted to ask about unpublished or un-indexed data, and this yielded a number of additional datasets. Archives of Ethiopian national journals were hand searched and articles related to the subject were included.

Cross-sectional studies were included, but health facility based surveys were excluded. If in a given area, multiple surveys had been conducted at different times, each was included in the analysis. From each study, the design, study population, method of diagnosis, date and location of survey, age range of sampled individuals, and number of individuals examined and diagnosed with podoconiosis were abstracted. Longitude and latitude of the survey locations were identified through a range of methods, including online search with GeoNames (www.geonames.org) or Wikipedia (www.wikipedia.org). For recent data from lymphatic filariasis mapping, the geographical positioning system (GPS) information relating to each location was obtained from the investigators.

### Satellite Driven Data

Previous studies suggest the importance of temperature, altitude and annual rainfall in the formation of irritant clay soil [2]. The Normalized Difference Vegetation Index (NDVI) is also considered to be associated with weathering of soil. Among other environmental characteristics, two were used: texture of the soil (classified as coarse, medium or fine), and the topography of the land. The topography of the land was obtained from University of Bern Centre for Development and Environment thorough the Ethiopian GIS society (http://www.cde.unibe.ch/Pages/Publication/1431/Default.aspx). The altitude, mean Land Surface Temperature (LST) and precipitation at 30-arcsecond (1 km) resolution were downloaded from the WorldClim website for the period 1950–2000 at 1 km spatial resolution [38]. Monthly average precipitation data at 1 km resolution for the period 1950–2000 and the mean annual precipitation was obtained. These climate variables were produced from global weather station temperature records gathered from a variety of sources for the period 1950–2000 and interpolated using a thin-plate smoothing spline algorithm [39]. The digital soil map of the world (Land and Water Development Division, FAO, Rome) at a nominal scale of 1∶5,000,000 was downloaded from the GeoNetwork website [40].

### Data Analysis

The prevalence and GPS data were entered using a Microsoft Excel 2007 spreadsheet and exported into STATA 11.0 (Stata Corporation, College Station, TX, USA). Point prevalence maps were developed in ArcGIS 10. Data for each location were extracted from the raster maps of each of the environmental variables.

Associations between prevalence of podoconiosis and individual environmental variables were investigated using the non-parametric Spearman’s rank correlation. The association between environmental variables and prevalence was further assessed using binomial logistic regression. Variables contributing to the model based on the log likelihood ratio test were retained in the final model. P-values <0.05 were considered significant. Statistical analyses were performed using STATA. All variables except topography (slope) and NDVI were treated as categorical variables. We have explored for linearity of the variables and in most cases non-linearity was observed which lead to categorizing them. The categories were based on expert opinion and previous observations in east Africa, where some cut-off points were indicated.

A multivariate regression model allowing for clustering was fitted using STATA 11 to assess the potential predictors of podoconiosis prevalence. The associations between podoconiosis prevalence, slope of the land (as surrogate for topography), altitude, mean annual LST, annual precipitation, mean annual rainfall, standard deviation of NDVI, and soil texture were examined. For each predictor, the Odds Ratio (OR), 95% confidence interval (CI), and p-values were recorded. Co-linearity was checked between all possible pairs of potential environmental variables; altitude and mean LST had a correlation coefficient >0.9, and since the mean LST has more to do with weathering of soil, altitude was excluded from the final model. All other variables were entered into the model adjusted for each other.

## Results

### Characteristics of the Survey Data

Ten studies conducted between 1969 and 2012 included data for 401,674 individuals older than 15 years of age from 229 locations (data points) in 9 of the 11 regional states of Ethiopia. Most (101, 44.1%) of the data points were from Oromia, the largest regional state in the country in terms of geographical area and population, 50 (21.8%) were from Southern Nations Nationalities and Peoples Region (SNNPR), and 24 (10.5%) from Amhara. There was no information for Addis Ababa or Somali (Table 1). The 229 data points were from 190 *woredas* (districts), of which 80 (43.1%) were within Oromia and 47 (24.7%) within SNNPR. Based on these results, the average prevalence of podoconiosis in Ethiopia was estimated to be 3.4% (95% CI: 3.3 to 3.4). The prevalence of podoconiosis varied by region: 4.8% in SNNPR, 4.4% in Harari, 3.0% in Amhara, 2.5% in Oromia, 1.6% in Tigray, 0.6% in Gambella, 0.4% in Benishangul Gumuz and 0.4% in Dire Dawa.

**Table 1:** Studies included in producing a podoconiosis map of Ethiopia.

Publication year	Place of study	Number of individuals sampled (prevalence)	Data points	Type of survey
1969 [15]	Multiple sites	247,908 (2.72%)	56	Market survey (visible leg swelling)
1973 [26]	Multiple sites	13,138 (2.77%)	25	School enquiry
1987 [25]	Ocholo, Southwest Ethiopia	2689 (5.4%)	1	Community based (physical examination)
1992 [21]	Gera & Didessa, Western Ethiopia	416 (7.5%)	2	Community based (physical examination)
1997 [24]	Pawe Northwest Ethiopia	1900 (7%)	1	Community based (physical examination and microscopic examination of midnight sample)
2003 [16]	Wolita zone, Southern Ethiopia	33,678 (5.46%)	7	Community based (physical examination)
2011 [17]	Gulisso west Ethiopia	38,420 (5.0%)	1	Community based (physical examination)
2012 [18]	Midakegni	1,656 (7.4%)	1	Community based (physical examination)
2012 [19]	Debre Elias and Dembecha Northern Ethiopia	50,620 (3.3%)	2	Community based (physical examination)
2012 [43]	Western Ethiopia in 112 Woredas	11,249 (4.6%)	133	Community based (ICT card)
Total		401,674 (3.4%)	229	

### Geographical Distribution of Podoconiosis

**Figure 1:**
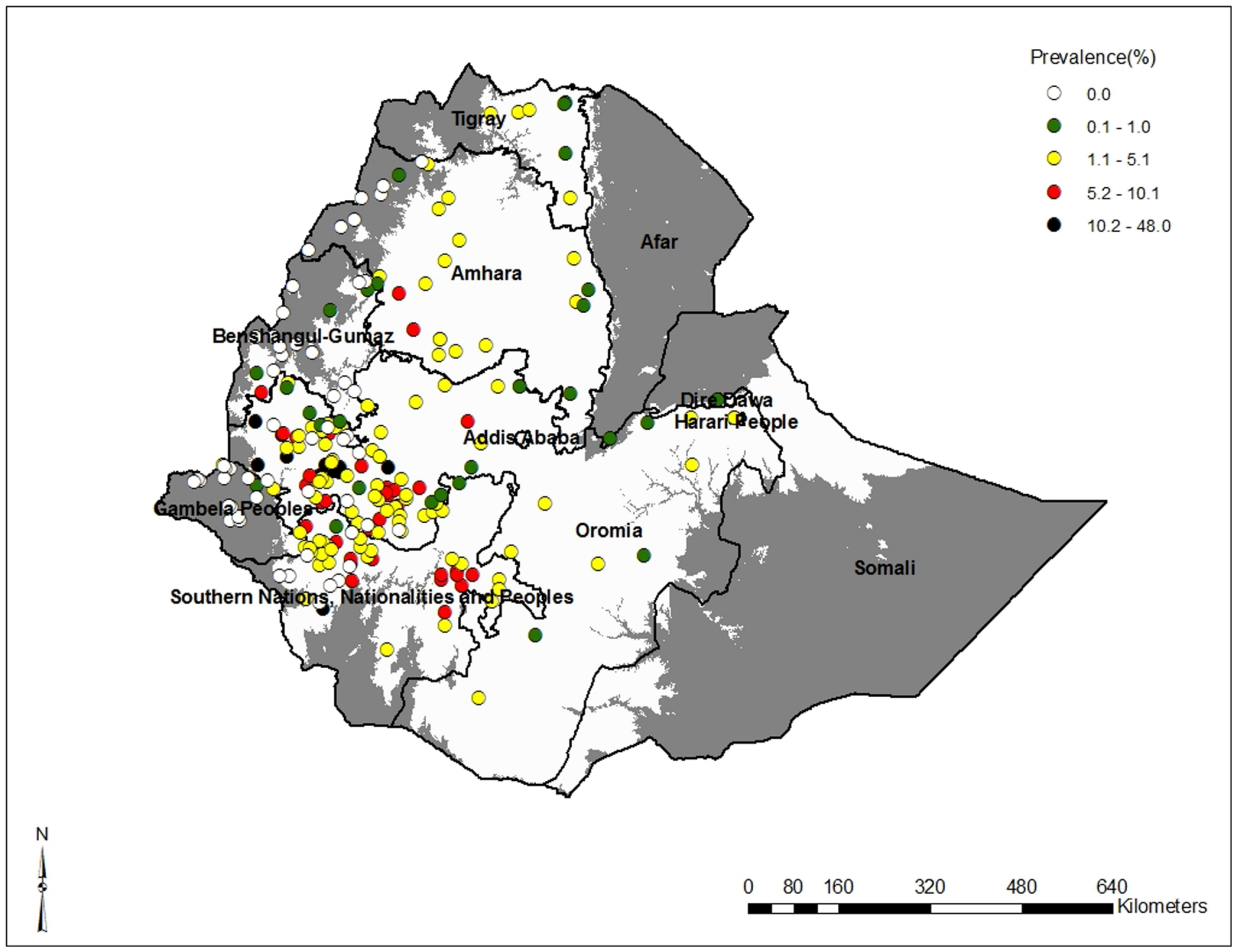
Geographical distribution of podoconiosis in Ethiopia, results from historical maps and recent surveys conducted on podoconiosis. In total 229 locations were identified with podoconiosis prevalence figures, and were geo-referenced. Circles indicate prevalence in villages and markets surveyed. Grey areas indicate areas where either altitude is below 1225 meters above sea level or mean annual rainfall is <900 mm or annual mean land surface temperature >24°C.

Figure 1 shows the geographical distribution of podoconiosis. There was wide geographical variation of prevalence from 0.0% to 48.0%. High prevalence of podoconiosis occurred in the southwest and southern parts of the country. The highest prevalence was recorded in Kelem Welega and Illubabor zones of Oromia. The prevalence of podoconiosis was generally lower in the far western part of the country, such as Gambella. Except for one location (with zero prevalence) no information was available about the occurrence of the disease in Afar and Somali (Table 2).

**Table 2:** Distribution of surveys included in the analysis, by region.

SN	Region	Number of survey points
1	Tigray	7
2	Afar	1
3	Amhara	24
4	Oromia	101
5	Somali	0
6	SNNPR	50
7	Dire Dawa	1
8	Addis Ababa	0
9	Benishangul Gumuz	22
10	Harari	2
11	Gambella	21
	**Total**	**229**

Based on the analysis of 190 *woredas* included in the 9 regional states, 39 *woredas* had zero prevalence, 104 a prevalence of 0.01–5.0%, 32 a prevalence of 5.1–10.0%, and 15 a prevalence of ≥10.0%. Of those *woredas* with a prevalence >5.0%, 27 (57.4%) were in Oromia and 16 (34.0%) in SNNPR; almost all (13 out of 15) *woredas* with a prevalence >10.0% were in Oromia (Table 3).

**Table 3:** Prevalence distribution of podoconiosis by *woreda* and region.

Region	Number of *woredas* by prevalence of podoconiosis					Total
	0.0%	0.01% to 1.0%	1.1% to 5.0%	5.1% to 10.0%	>10.0%	
Afar	1	0	0	0	0	1
Amhara	3	3	14	2	0	22
Benishangul Gumuz	13	3	3	1	0	20
Gambella	8	1	3	0	0	12
Harari	0	0	1	1	0	2
Dire Dawa	0	1	0	0	0	1
Oromia	7	6	40	14	13	80
SNNPR	7	1	23	14	2	47
Tigray	0	2	3	0	0	5
**Total**	**39**	**17**	**87**	**32**	**15**	**190**

### Environmental Variables Associated with Podoconiosis

Areas with zero prevalence were characterized by a mean altitude of 1055 masl, mean annual rainfall of 1293 mm, topography of 2.1 degrees and temperature of 24°C. High prevalence areas (>5%) were characterized by mean altitude >1600 masl, temperature between <20.5°C, mean annual rainfall >1500 mm and mean annual precipitation >130 mm (Table 4).

**Table 4:** Environmental characteristics of different categories of prevalence of podoconiosis.

Environmental variable	Prevalence classification			
	0%	0.1%–5.0%	5.1%–10%	>10.0%
Mean altitude (masl)	1055.2	1820.6	1804.8	1685.3
Mean annual rainfall (mm)	1292.9	1407.7	1592.6	1638.5
Mean slope of the land (°)	2.1	2.1	2.9	1.8
Mean annual temperature (°C)	23.8	19.3	19.4	20.2
Mean annual precipitation (mm)	107.7	117.2	132.0	136.0

Correlation of the observed geographical distribution of podoconiosis with selected large-scale environmental variables was investigated. Absence or very low prevalence of podoconiosis was observed in areas with annual total rainfall <900 mm (p = 0.36, *p<0.001)*, and in areas with annual mean LST >24°C (p = −0.35, *p<0.001).* Although difficult to indicate a clear cutoff, there was a positive correlation between annual mean precipitation and prevalence (p = 0.36, *p*<0.001).

**Figure 2:**
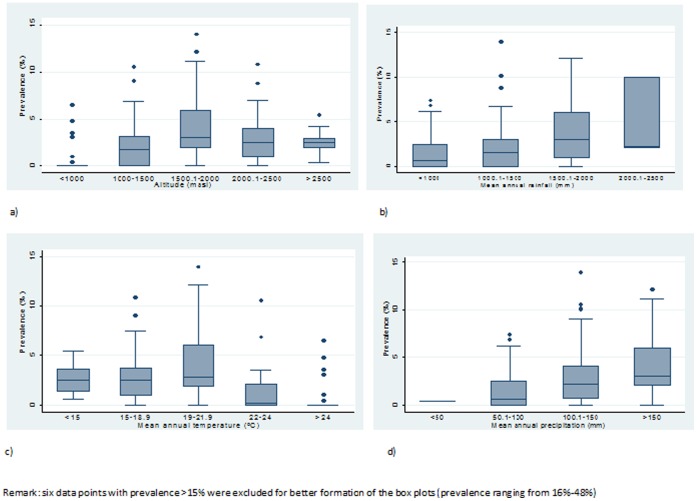
Box plots of environmental variables and prevalence of podoconiosis. a) altitude in meters versus prevalence of podoconiosis b) annual rainfall in mm versus prevalence of podoconiosis c) Mean annual Land Surface Temperature and prevalence of podoconiosis and d) Annual precipitation versus prevalence of podoconiosis.

Possible associations of environmental variables previously documented to be associated with podoconiosis were identified and associations were checked using box plots (Figure 2). There was a clear trend of increasing prevalence as altitude and rainfall increased. The possible association of environmental variables and prevalence of podoconiosis was modelled. The final model showed that a one-unit increase in slope was associated with an increase of approximately 16% increase in the podoconiosis prevalence (OR = 1.16; 95% CI = 1.15 to 1.17; p<0.001). Compared to points with annual precipitation ≤50 mm, those areas with precipitation >50 mm had higher odds of high prevalence podoconiosis (OR = 8.11, 95% CI = 5.85 to 11.25; p<0.001). Compared to areas with annual mean LST ≤24°C, those areas with mean annual LST >24°C had lower odds of high prevalence of podoconiosis (OR = 0.48, 95% CI = 0.41 to 0.57). Compared to medium size soil texture, areas with fine texture had higher odds of high prevalence podoconiosis (OR = 1.09, 95% CI = 1.03 to 1.16) (Table 5).

**Table 5:** Output of binomial logistic regression of environmental variables and prevalence of podoconiosis, 229 locations in Ethiopia.

Variable	Category	Unadjusted Odds Ratio	AOR 95% Conf. Interval	P>|z|
Precipitation (mm)				
	≤50	1.00	1.00	
	>50	8.25 (5.97 to11.39)	8.11 (5.85 to 11.25)	<0.001
Mean annual Land Surface Temperature (°C)				
	≤24	1.00	1.00	
	>24	0.40 (0.34 to 0.47)	0.48 (0.41 to 0.57)	<0.001
Mean annual rainfall (mm)				
	≤1000	1.00	1.00	
	>1000	1.21 (1.14 to 1.27)	0.97 (0.92 to 1.03)	0.436
Altitude (masl) €				
	≤1225	1.00		
	>1225	3.06 (2.60 to 3.59)		
Soil texture				
	Fine	1.08 (1.02 to 1.15)	1.09 (1.03 to 1.16)	0.002
	Medium	1.00	1.00	
Topography of the land(°)		1.17 (1.15 to 1.18)	1.16 (1.15 to 1.17)	<0.001
SD of NDVI§		1.00 (0.99 to 1.00)	0.99 (0.99 to 1.03)	0.192

§ SD = standard deviation, NDVI = Normalized Difference Vegetation Index, masl = Meters above sea level, mm = millimeter, € Co-linearity was checked between all possible pairs of potential environmental variables: altitude and mean LST had a correlation coefficient >0.9, and since the mean LST has more to do with weathering of soil, altitude was excluded from the final model.

## Discussion

Podoconiosis is gradually attracting global attention [41], [42], and as the profile of the disease increases, understanding the geographical distribution and environmental factors affecting this distribution will become important. Identifying the environmental correlates of disease distribution is the first step to producing risk and distribution maps to guide decision making for interventions. Although podoconiosis has been described in Ethiopia for four decades [26], [27], the current disease burden and geographical distribution is not known. Here we summarize its distribution based on historical and contemporary data, and identify environmental variables associated with the distribution of the disease. (Figure S3).

The prevalence of podoconiosis in Ethiopia was found to be 3.4%, which is higher than the 2.7% reported by Ooman in 1969 [15] in his market-based survey of 56 sites or the 2.8% reported by Price from school-based surveys conducted in 1974 [26]. It is also higher than the prevalence documented in Burundi (1.0%) and in Rwanda (0.6%) in the 1970s [14]. More recent studies included in the current analysis have indicated prevalences of 5.5% in Wolaita, southern Ethiopia [16], 5.4% in Ocholo [25], southwestern Ethiopia, 7.0% in Pawe [24], 7.5% in Gera and Didessa [21] and 5.2% in Gulisso [17], western Ethiopia, and 7.4% in central Ethiopia [18]. Absence of information from Addis Ababa, Afar and Somali regions and the diagnostic methods used reduces the precision of the estimate. In addition, one of the largest surveys included in this review was based on purposive sampling covering areas that were thought to be endemic for podoconiosis, which might result in over estimation of the prevalence.

There was significant geographical variation within Ethiopia; high regional prevalences were recorded in SNNPR, Oromia and Amhara. These are consistent with findings from individual studies and environmental characteristics studied. All of the areas with prevalence >10% were found to be non-endemic for LF (ICT positivity of 0%) [43]. These districts are located in Illubabor and Kelem Welega zones of Oromia, and these areas were already identified by civil society organizations as endemic for podoconiosis.

In this analysis, we found associations between prevalence and temperature, altitude, rainfall, precipitation, topography of the land and soil texture. Previous studies have documented association of temperature, altitude and rainfall with occurrence of podoconiosis in East Africa [2]. High prevalence of podoconiosis was documented in areas with altitude of >1500 masl, mean annual rainfall of >1500 mm and mean annual LST of 19–21°C. Altitude governs temperature and rainfall, and these affect weathering; hot and humid environments increasing weathering and type of soil produced. In arid regions, the lack of precipitation inhibits chemical weathering, leading to coarse textured soil [36], [37]. The topography of the land increases water runoff and thus weathering of rocks [36], [37] and in the present study, was also associated with podoconiosis prevalence. Soil texture was also associated with prevalence of podoconiosis, confirming an earlier study which documented an association between particle size and occurrence of podoconiosis [44].

Absence of podoconiosis was associated with mean annual rainfall <900 mm, mean altitude of 1225 masl and mean annual temperature of >24°C. Previous studies in east Africa documented the absence of podoconiosis in areas at ≤1000 masl and with annual rainfall ≤1000 mm. These findings might justify exclusion of areas below these environmental limits in future mapping activities [2]. Based on these findings Afar and the majority of Somali regional state should be classified into the ‘no podoconiosis’ category. This is in agreement with the expert opinion of two Ministry of Health Regional Health Bureaus, who state that podoconiosis is absent in their regional states (Nebiyu Negussu, Somali Regional Health Bureau and Aregawi Gebremedhin, Afar Regional Health Bureau, personal communications). Of note is that lymphatic filariasis was recently documented in areas up to 1698 masl [43] indicating that there are potential areas between 1225 and 1698 masl where filariasis and podoconiosis co-occur – clearly, this must be taken into account when mapping either disease. In areas of possible overlap between the two diseases, anti-filarial antibody examinations may be required [45], [46] as well as entomological studies of the vector population [47]. The best strategy in countries where both LF and podoconiosis exist is coordinated mapping: both diseases have similar clinical features and differential diagnosis will have to be made, and both have the same population group for mapping (i.e. individuals >15 years old).

There is no systematic, large scale podoconiosis control program in Ethiopia. For over 20 years, podoconiosis treatment and prevention has been offered on a small scale by a range of community based organizations and international non-governmental organizations. Recently, podoconiosis was recognized by the Ministry of Health as one of the eight national priority NTDs, and was included in the National Master Plan for NTDs (2013–2015). With this inclusion, and growing interest from the regional health bureaus, it is likely that the treatment and prevention of podoconiosis will be scaled up in Ethiopia. By describing the geographical variations of podoconiosis within Ethiopia, we hope to characterize areas in which prevention and treatment should be targeted. The rainfall, altitude and temperature limits of podoconiosis will help to exclude areas in which the disease is unlikely to be a significant public health problem, and so will enable limited resources to be focused on priority areas. We are planning to conduct nationwide mapping of podoconiosis in Ethiopia and the results presented here have implications for this exercise. For example, the majority of districts in Afar, Gambella, Somali and part of Benishangul Gumuz are characterized by the environmental features associated with zero prevalence.

There are limitations to the current work; the surveys included used different methods, and the case definition of podoconiosis across the studies was inconsistent. The two largest surveys included in this analysis used market count and school enquiry techniques, both of which may underestimate prevalence. However, the investigators validated the surveys by comparing with community based censuses, and demonstrated good agreement [27]. In the older map developed by Price [26] some of the locations do not have precise estimates of prevalence, so the average of the lower and upper boundaries was taken. The studies included in the analysis are over two generations; there may be changes in shoe wearing practices over time affecting the prevalence of podoconiosis. Nonetheless, repeated surveys [16], [19] in some areas suggest that there have been no significant changes in prevalence of podoconiosis over the last 40 years. The other limitation of the analysis is that most of the surveys did not use a diagnostic test, relying on observations or physical diagnosis. Only three used any biomedical test to enable differential diagnoses of conditions with a similar symptomatology to podoconiosis (e.g. lymphatic filariasis). Nonetheless most of the studies were conducted in areas potentially endemic for podoconiosis based on geographical parameters. In addition we have used data from recent LF mapping to exclude areas endemic for LF from the analysis. Rapid diagnostic tests for lymphatic filariasis are now available, and will decrease misclassification in areas where these two diseases overlap. The lack of information from three regional states of Ethiopia is also another limitation, nonetheless only 12% of the population lives in these three regional states. In addition, Addis Ababa is the capital city, and shoe-wearing is almost universal. The upcoming mapping projects will address the above mentioned limitations and will generate more reliable estimates.

### Conclusion

Our analysis sheds light on the ecology of podoconiosis in Ethiopia, and shows that podoconiosis only represents a public health problem of major concern under certain environmental conditions, presumably those conditions favourable for the weathering of rock to produce specific types of soil. The disease is highly prevalent in the highlands of Ethiopia particularly in the southern and south central areas. Climatic conditions, primarily altitude, rainfall, precipitation and temperature, influence the weathering of rocks and determine the type of soil generated, which in turn probably influences the distribution of podoconiosis. High prevalence areas are characterized by mean altitude >1500 masl, temperature between 19–21°C, mean annual rainfall >1500 mm and mean annual precipitation >130 mm. Identifying other variables influencing prevalence might be achieved through inclusion of high resolution geological and soil maps in future analyses. Deciding on a threshold for classification of districts as ‘endemic’ or ‘non-endemic’ for podoconiosis will be a priority in targeting intervention to where the disease is prevalent. Finally, this is work in progress and the results presented here will be refined in future work.

## Supporting Information

Figure S1 Podoconiosis at different stages in four approximately 50 year old women, from Ethiopia, (Picture by Gail Davey).
(TIF)

Figure S2 A barefooted farmer ploughing a red clay soil field using the traditional method pulled by two oxen (Picture by Gail Davey).
(TIF)

Figure S3 Women in northern Ethiopia walking barefoot (Picture by Kora Image, Abate Damte).
(TIF)
