# Supplementary figures and images for: Spatial Distribution of Podoconiosis in Relation to Environmental Factors in Ethiopia: A Historical Review
Source: PLoS One. 2013 Jul 9;8(7):e68330. doi: 10.1371/journal.pone.0068330 (PMC3706425; doi:10.1371/journal.pone.0068330)

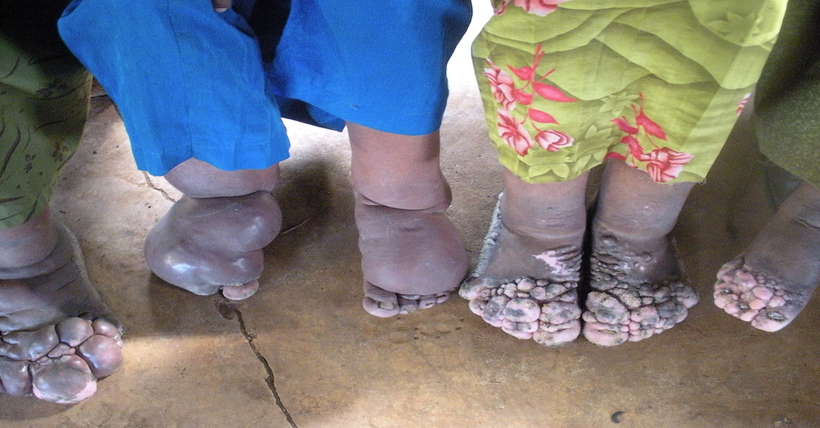

Supplement: Figure S1 — Podoconiosis at different stages in four approximately 50 year old women, from Ethiopia, (Picture by Gail Davey). (TIF) [file pone.0068330.s001.tif]

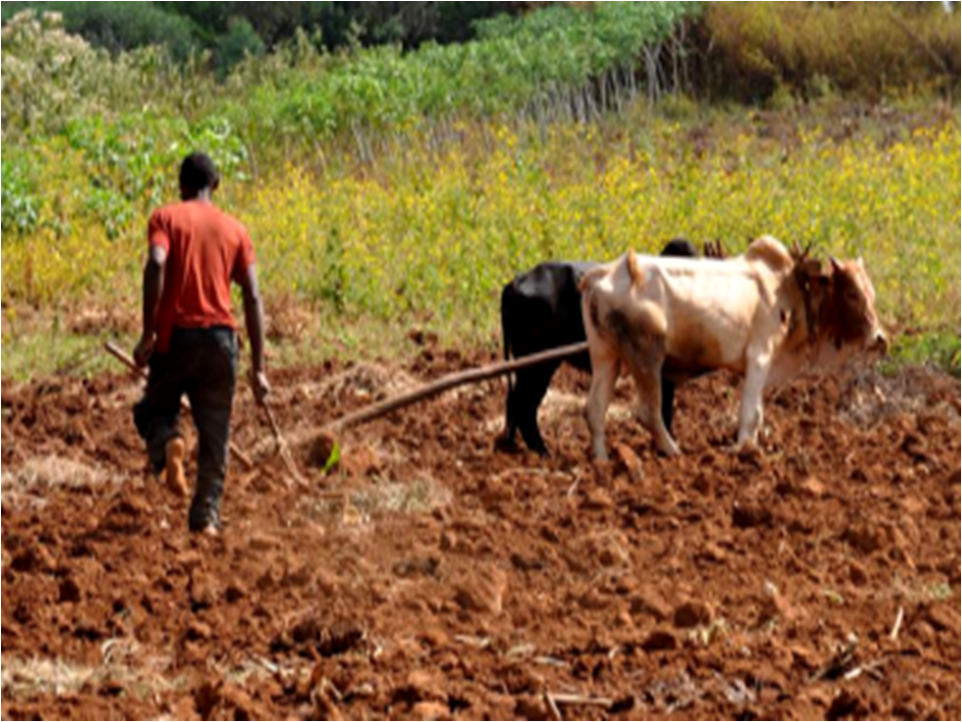

Supplement: Figure S2 — A barefooted farmer ploughing a red clay soil field using the traditional method pulled by two oxen (Picture by Gail Davey). (TIF) [file pone.0068330.s002.tif]

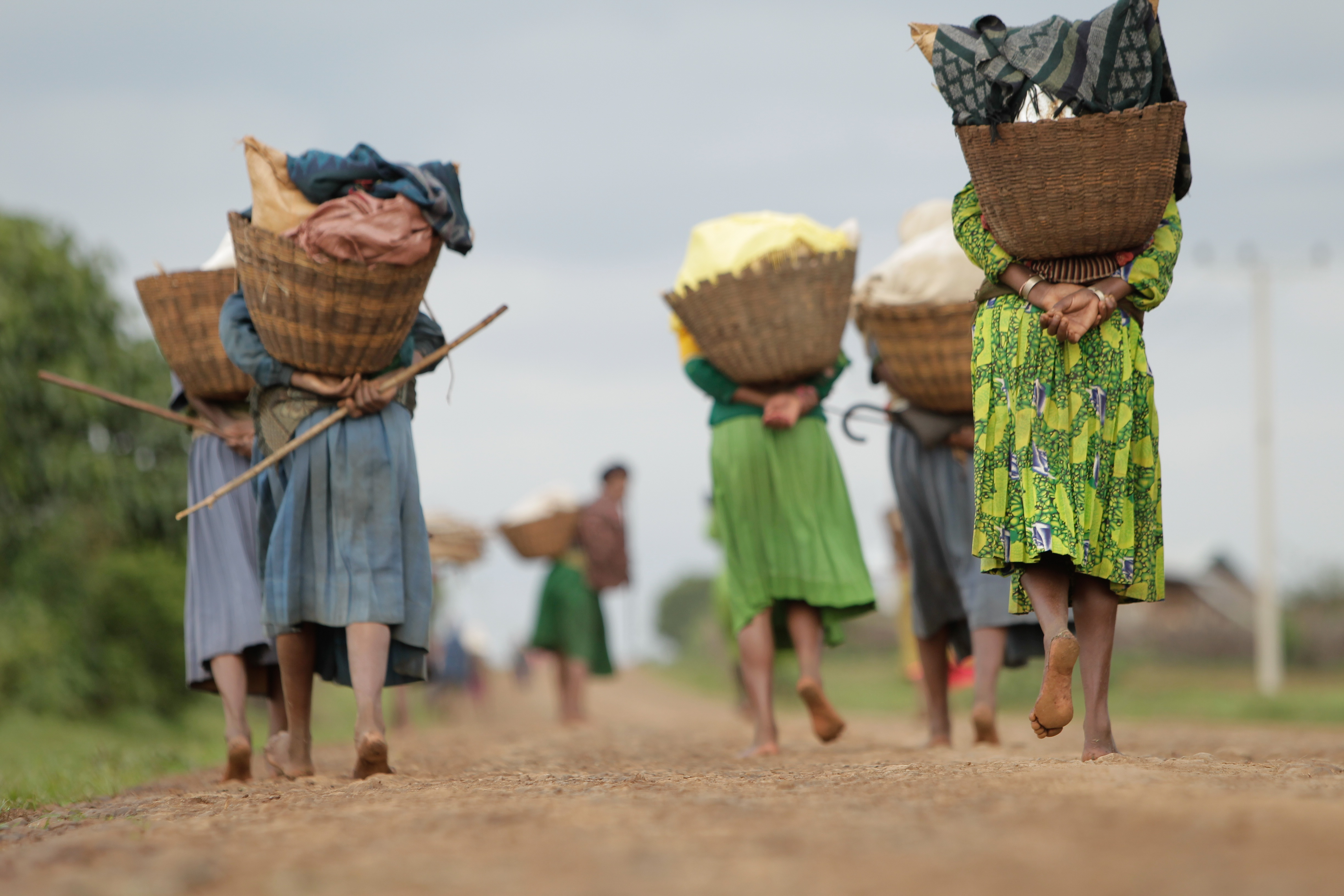

Supplement: Figure S3 — Women in northern Ethiopia walking barefoot (Picture by Kora Image, Abate Damte). (TIF) [file pone.0068330.s003.tif]
